# Patient perspectives on the British Columbia Biosimilars Initiative: a qualitative descriptive study

**DOI:** 10.1007/s00296-021-04874-8

**Published:** 2021-05-07

**Authors:** Caitlin Chew, Magda Aguiar, Nick Bansback, Michael R. Law, Mark Harrison

**Affiliations:** 1 Faculty of Pharmaceutical Sciences, The University of British Columbia, Vancouver Campus, 4625-2405 Wesbrook Mall, Vancouver, BC V6T 1Z3 Canada; 2 School of Population and Public Health, The University of British Columbia, 2206 East Mall, Vancouver, BC V6T 1Z3 Canada; 3 Centre for Health Evaluation and Outcome Sciences, St. Paul’s Hospital, 588-1081 Burrard Street, Vancouver, BC V6Z 1Y6 Canada; 4 Centre for Health Services and Policy Research, The University of British Columbia, 201-2206 East Mall, Vancouver, BC V6T1Z3 Canada

**Keywords:** Biosimilars, Switching, Biosimilar switching, Non-medical switching

## Abstract

In May 2019, the Government of British Columbia (BC) announced the implementation of the Biosimilars Initiative, mandating the switch of biologic (originator) drugs to biosimilars for certain patient populations in the hopes of optimizing public resources. Through this qualitative study, we aimed to identify patients’ perspectives as they undergo this change. From October 2019 to July 2020, we conducted nine pre- and six post-switch to biosimilar interviews with BC, English speaking participants, who were 18 years or older, and were currently taking a biologic medication. Participants were interviewed pre- and post-switch to a biosimilar medication and interviews were audio-recorded and transcribed verbatim for qualitative analysis. Interviews were thematically analysed and major themes and sub-categories were elucidated. The themes derived from pre and post-switch interviews captured participants’ anticipated or experienced barriers and enablers to the policy change. In general, the fears and apprehension of participants approaching the switch, including concerns surrounding the efficacy and safety of biosimilars, were addressed by their rheumatologist and social support circles. For the most part, participants were able to successfully manage their disease regardless of their baseline concerns about efficacy and safety. Experiences of changes in health delivery models were also observed secondary to the impact of the COVID-19 pandemic amongst participants. This study is the first of its kind to characterize the patient perspective regarding the BC Biosimilars Initiative. The incorporation of the patient perspective, including adequate provider-patient communication and shared decision-making can help to inform future non-medical switching policy changes.

## Introduction

Biologics are a type of medication therapy made of large, complex molecules that are engineered from living organisms such as live yeasts and bacteria [1]. The first version of a biologic developed is known as an originator drug. Due to an array of reasons, including the complex nature of biologic molecules and the proprietary nature of the biologic production processes, they cannot be directly replicated. Biosimilars are molecules that are based on biologic medications that though not entirely identical to the originator drug, are assumed to have the same therapeutic characteristics of the originator drug [2]. This issue, however, is not unique to biosimilars; originator biologics cannot be replicated exactly so there is variability between batches of biologics and over time [3, 4].

The use of biologics in patients with autoimmune conditions has been shown to lead to improvements in patients’ health-related quality of life [5–7]. Specifically, the positive impact of biologic drugs on clinical outcomes of disease management and their significant role in slowing down disease progression, particularly for rheumatic diseases, has been characterized in the past [8]. Nevertheless, the effectiveness of this class of drugs comes at a cost, with biologics consistently listed among the classes of drugs accounting for the highest proportion of total drug spending in Canada [9, 10]. In 2018, biologic drugs including etanercept (Enbrel) and infliximab (Remicade), which have biosimilar equivalents, contributed to a total of $125 million of British Columbia’s (BC) drug expenditure [11]. Presently, biosimilars are thought to cost 25% to 50% less than their originator drug, and the potential cost savings from the use of biosimilars in Canada by 2021 have been estimated to be as high as $842 million [12, 13]. To date, there has been a lack of biosimilar uptake in Canada and the United States, suggesting that these potential cost-savings have not yet been realized [10, 14].

To reduce the economic burden of biologics, the BC government announced Phase 1 of the “Biosimilars Initiative” in May 2019, expanding the use of biosimilars for particular medical indications including ankylosing spondylitis, rheumatoid arthritis, psoriatic arthritis, and plaque psoriasis. With the implementation of the “Biosimilars Initiative” this policy mandates that patients on specific biologic medications, including etanercept (Enbrel) and infliximab (Remicade) for ankylosing spondylitis, rheumatoid arthritis, and plaque psoriasis would be switched to a biosimilar equivalent by November 25, 2019 [15]. In BC, by March 2020, 78% of patients (almost 12,000) had transitioned from biologic to biosimilars [16]. This policy change was the first of its kind in Canada and North America [15].

With the novelty of this policy change in the province and country, the objective our study was to characterize patients’ expectations, concerns, and perceptions ahead of switching from originators to biosimilars followed by their experience of mandatory switching. We anticipated that patients would be anxious about the switch and reluctant to change to biosimilars for non-medical purposes, but that if the biosimilars were (as evidence suggests) equivalent, that their perspectives would change post-switch.

## Methods

### Study design

We conducted semi-structured interviews, in-person or by telephone, with participants with rheumatic disease prior to and post-switch from a biologic medication to a biosimilar. To maximize consistency, all interviews were conducted by one researcher (MA) who has had previous experience conducting interview-based research. Using convenience sampling, participants were recruited from two rheumatology clinics in BC and email invitations were sent to potential participants. The number of participants recruited were based on convenience sampling and not thematic saturation. Participants were recruited by their rheumatologist, with the support from the practice’s administrative staff, who directly contacted participants who were scheduled to switch and scheduled the interviews. The inclusion criteria included English speaking individuals who are aged 18 years or older, are currently taking a biologic drug affected by the BC Biosimilars Initiative and were scheduled to be switched to a biosimilar. Before the interviews, participants were asked to fill out a demographic questionnaire and consent form. The interview prior to their switch consisted of open-ended questions about the patients’ general perceptions of the change, their baseline knowledge of their medications that are affected by the policy change, and any concerns they had about the anticipated impact the switch may have on their disease management. At the first interview, patients consented to be contacted for a second interview following their switch to a biosimilar at a date to be scheduled later. Post-switch, participants were asked open-ended questions regarding any changes in perception to the policy change and any shifts in expectations and opinions post-switch. Example interview questions and prompts are presented in Table 1. In light of the COVID-19 global pandemic declared by the World Health Organization in March 2020, questions about the impact of the pandemic on participants’ switch to biosimilars were also included in these interviews as their switch spanned the timeline of the global pandemic [17].

**Table 1:** Examples of questions and prompts from re and post-switch interviews

Pre-switch		Post-switch	
Example questions	Example prompts	Example questions	Example prompts
What do you know about the switching policy and upcoming transition from originators to biosimilar drugs in BC? What do you think are the impacts of switching from originators to biosimilars?	Do you feel that you understand the reason for the change? Are there specific benefits or harms that you foresee arising from transitioning to biosimilars?	What has the biosimilar experience been like for you? What have the impacts of switching from originators to biosimilars been for you? Has the current COVID-19 global health pandemic affected or impacted your switch?	Do you feel like any of your prior expectations or opinions regarding the switch have remained the same or changed? How has your overall disease management been like throughout the switch?

### Data analysis

Data collected from the interviews were audio-recorded and transcribed verbatim. Both pre-and post-switch interviews were coded line-by-line, inductively, using an iterative, thematic approach, guided by the overarching research question. The preliminary analysis was conducted by one research investigator (CC). The derived codes were continuously compared and contrasted by all research investigators to identify sub-categories and to elucidate final major themes. Qualitative analyses of these interviews were conducted using NVivo 12 (QSR International). This study was approved by University of British Columbia Behavioural Research Ethics Board (H19-02169).

## Results

From October to November 2019, we interviewed a total of nine participants prior to their switch from biologics to biosimilars, and in July 2020, we re-interviewed a total of six participants post-switch to biosimilars. When invited to participate in a second, follow-up interview, three remaining participants could not be contacted to schedule a follow-up interview. The average age of participants was 60.7 (range: 47–80) with 67% (*n* = 6) of respondents aged 65 years or less. The majority of the respondents were of European descent (89%; *n* = 8). Participants were based in two geographical health regions in BC. Additional participant characteristics are listed in Table 2.

**Table 2:** Participant demographic data

Characteristic	All participants *N* = 9
Age	
Mean (range)	60.7 (47–80)
Age distribution no. (%)	
≤ 65 years	6 (67%)
> 65 years	3 (33%)
Gender no. (%)	
Female	7 (78%)
Male	2 (22%)
Race no. (%)	
European	8 (89%)
Indigenous	1 (11%)
Highest level of education no. (%)	
High school degree of equivalent	2 (22%)
Some college but no degree	3 (33%)
Bachelor’s degree	3 (33%)
Associate degree	1 (11%)
Range of total household income no. (%)	
$30,000–$39,000	2 (22%)
$70,000–$79,000	2 (22%)
$80,000–$89,000	2 (22%)
$90,000–$99,000	1 (11%)
$100,000 or more	2 (22%)

### Pre-switch interviews

Thematic analyses of the pre-switch interviews identified four major themes: (1) impact of switch on disease management; (2) baseline knowledge of the policy change; (3) perceived enablers to the switch; and (4) perceived barriers to the switch. These major themes, sub-categories, thematic descriptions, and corresponding sample quotations are listed in Table 3.

**Table 3:** Sample participant quotes for major themes and sub-themes prior to biosimilar switch

Major themes, conceptual categories, and corresponding quotes		Sub-theme explanations
Theme 1A: impact of switch on disease management		
Maintenance of disease control	*“Hopefully, it sounds like it should just be smooth, and just I won’t feel, like, I don’t feel anything now with my arthritis. Hopefully, that will just continue.”*	Participants shared concerns surrounding anticipated changes to disease control during their switch
Logistics of switch	*“So now what happens? Does the biosimilar have a similar type of auto-injector, or does it even have an auto-injector? I don’t know any of that.”*	Participants were concerned with potential changes to scheduled rheumatologist visits, the transition period between medications, and alterations to the mode of drug delivery
Impact on quality of life	*“You have a nice, quiet life going. Everything is working well and now we’re going to switch drugs. Who knows what’s going to happen? And maybe it’s not warranted, but who knows?”*	Participants expressed concerns over negative impact on quality of life when making the switch
Theme 2A: baseline knowledge of policy change		
Information sources	*“I saw it on TV, and then I got notified in the mail from, both my benefits company and Pharmacare sent me a letter saying that as of November something, you weren’t, you’d have to switch.”*	Participants gained knowledge about the policy change through self-research and through healthcare providers
Government policy making	*“It’s the government that’s, now, I don’t know if this is because, in my opinion, I think they’re just doing it to save money, and obviously, that’s the reason. It’s the same type of drug, just made by a different company.”*	Participants understood the rationale behind the policy change, namely, the government cost savings associated with biosimilars
Biosimilar generation	*“Yeah, I believe so. I mean, the government is paying a lot of money for the original drug, and there were really no, there was a patent on it, yes, but there weren’t any, you know, the biosimilars that are similar to the—what they call them?—the generic drugs, right?”*	Participants had a good grasp of the similarities and differences between biologics and biosimilars
Theme 3A: perceived enablers to the switch		
Financial enablers	*“It’s cheaper for people.”*	Lower cost associated with biosimilars was a motivating factor for participants
Support systems	*“[My son] is convincing me it’s gonna be okay.”*	The presence of support systems, both from family and healthcare providers, were perceived as enablers to their switch
Positive outlook	*“I’m convinced that it’ll be okay, and I was very lucky when I first started on Remicade. I didn’t have any problems, and I was able to go on the hour, it used to be a 2 h time, and now they could put me on 1 h quite quickly because I didn’t have any problems or allergies or reactions to it. I had such a positive experience because when I was put on it, I was quite debilitated and in a lot of pain. Within 24 h, I felt so much better, so I was out snowshoeing with friends, and we were all screaming and dancing. It was so exciting, and the difference was quite profound.”*	General positive outlook and optimism was expressed by participants prior to their changeover
Acceptance of change	*“Maybe it’s the older I get, I’m not just going to go along with things for the sake of going along with things. Show me why it’s beneficial for me or, in the larger context, why it’s beneficial.”*	Participants were able to use perceived enablers of the switch to accept the policy change
Theme 4A: perceived barriers to the switch		
Cost concerns	*“So we, my husband and I, we were thinking, ‘Okay. Now, are we gonna have to pay that deductible now?’”*	Participants expressed uncertainty surrounding the possibility of out-of-pocket payments secondary to the change
Drug properties	*“Well, I am hoping that it’s gonna work as well as the Enbrel, and no side effects because that’s always a big concern. Any time you switch, it’s not a guarantee that it’s going to work, so it can take you out of any kind of remission that you are in.”*	Participants shared concerns over biosimilar properties, including lack of efficacy and potential adverse effects compared to the originator drug
Negative outlook	*“No, I am not supportive of the change. It’s being forced upon me. If something is working, it shouldn’t be changed. It should be left alone.”*	Participants shared feelings of apprehension and displeasure towards the policy change
Resistance towards change	*“But I just feel frustrated that this is happening, that I have no, I have no say in this matter. It’s being decided for me.”*	Participants’ perceived barriers informs the resistance expressed towards the change

Many participants shared their anxiety surrounding the impact of the switch on disease management (Theme 1A) and their disagreement with the policy change expressing that “when you start switching drugs, you do not know the side effects or what’s going to happen” (P1-9). Participants spoke about the expected impact the policy change would have on their health-related quality of life, sharing previous challenging experiences when starting their biologics and their fear of these challenges re-emerging when switching over to the biosimilar. For the most part, participants had good baseline knowledge of the policy change (Theme 2A) and understood the differences and similarities between biologics and biosimilars. Generally, participants sought information and gained knowledge about the policy change through self-research, or from their healthcare providers such as their rheumatologist or pharmacist. Prior to their switch, participants understood the lower cost to the Government of BC associated with biosimilars, and the presence of healthcare and family support systems as well as their overall positivity towards the change acted as perceived enablers to the switch (Theme 3A). Overall, participants felt that they had the necessary information and resources regarding their health and medication management. Conversely, participants also shared perceived barriers to the switch (Theme 4A), notably, concerns over the potential differences in efficacy and safety profiles of biosimilars compared to biologic drugs. Many participants shared feelings of apprehension and displeasure towards the change. Cumulatively, these concerns and attitudes led participants to express frustration over this decision and a desire for more information and justification over the change.

### Thematic map of major themes from participants pre-switch

**Fig. 1:**
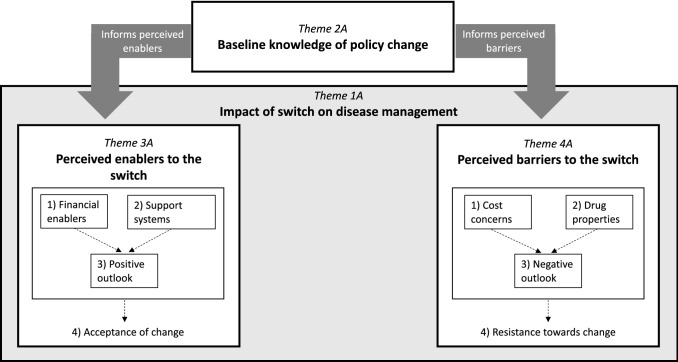
A thematic map pre-switch depicting how a patient’s baseline knowledge of the policy change (Theme 2A) informs both their perceived barriers (Theme 3A) and enablers (Theme 4A) of the switch. These themes are all encompassed by the overarching impact that the biosimilar switch has on patient disease management of their chronic condition (Theme 1A). Dashed arrows show relationships between sub-themes

Relationships between and within themes drawn from interviews pre-switch are depicted in Fig. 1. Baseline knowledge of the policy change (Theme 2A) informed both participants’ perceived enablers (Theme 3A) and barriers (Theme 4A) to the switch. Perceived enablers (Theme 3A), including financial motives and participant support systems, both contributed to participants sharing a positive outlook on the policy change. These drivers of change reinforced participants’ acceptance of the policy change. Conversely*, perceived barriers* (Theme 4A), including cost concerns and the biosimilars’ efficacy profiles, informed participants’ negative outlook on the switch. These factors contributed to participants’ expressed resistance towards the policy change. Both perceived enablers and barriers of the switch are encompassed by the overarching theme of impact of switch on disease management (Theme 1A).

### Post-switch interviews

The resultant themes from the post-switch interviews include: (1) preconceived ideas of switch; (2) experienced enablers of switch; (3) experienced barriers of switch; (4) support systems; and (5) effect of COVID-19 pandemic on switch. These themes, sub-categories, and corresponding example quotations are listed in Table 4.

**Table 4:** Sample participant quotes for major themes and sub-themes after their switch to biosimilars

Major themes, conceptual categories, and corresponding quotations		Sub-theme explanations
Theme 1B: preconceived ideas of switch		
Negative expectations of switch	*“I just didn’t know if it would work as well as the other medication I was taking”*	Participants reflected back on negative attitudes and opinions prior to the switch
Positive expectations of switch	*“My expectation was it was gonna be the same and things would just carry on”*	Participants reflected back on positive opinions and perceptions of the switch
Theme 2B: experienced enablers of switch		
Informed decision-making	*“In November, I went and saw my rheumatologist, and that, you know, for the purpose of just talking about it and doing the switch”*	Participants felt well-informed about their switch and their medication management through the support of their rheumatologist
Disease management	*“But I haven’t noticed anything, […] there’s been no more, no more stiffness or not anymore, anything more than I normally would expect at this time”*	Participants shared that they were able to successfully manage their disease throughout the course of the switch, with limited loss of disease control
Efficacy of biosimilar	*“I just didn’t know if it would work as well as the other medication I was taking. So that was my only concern, and it hasn’t been an issue”*	Participants shared that they had not noticed any changes in efficacy of their biosimilar and were able to continue to manage their medical condition
Limited adverse effects	*“It was quite a smooth transition”*	Participants communicated that they experienced limited adverse effects during the transition
Positive experience	*“Quite smooth [transition] actually, and it’s been quite good.”*	Overall, participants shared positive attitudes towards their experiences switching to biosimilars
Theme 3B: experienced barriers of switch		
Adverse effects	*“My stomach area to do the injection, and yet at least for a little time thereafter, it becomes uncomfortable wearing clothing over the injection site, just site tenderness. If I get involved with doing things, it’s gone within minutes.”*	Participants expressed experiences of adverse effects during the switch, namely, increase in pain or discomfort at the injection site
Drug packaging	*“It’s just the needle going in is more painful.”*	Participants hypothesized that the increase in adverse effects from the biosimilars could be attributed to the changes in needle type
Cost concerns	*“I never paid for anything with Enbrel. The first time I go in to fill a prescription, they ask me for $75 over and above my plan.”*	Participants expressed that their out-of-pocket payment for their biosimilars have remained unchanged or have increased secondary to the switch
Loss of disease control	*“I’ve had some flare-ups. I’ve been, I didn’t think it was working as good. I don’t think it works as good.”*	One participant expressed changes in drug regimen and loss of disease control during their transition
Desire to revert to originator drug	*“I wanted to stay on Remicade.”*	Their overall shared experiences led some participants to expressing the desire to stay on their biologic
Theme 4B: support systems		
Family support systems	*“Like, as far as going into stores and things like that, my husband’s been doing most of that.”*	The support from participant family members throughout the switch to biosimilars
Healthcare provider support	*“He answered the question beforehand. I mean, I asked him about the different biosimilars that were coming out. He said that he’d looked at studies in Europe and said that, for the type of arthritis that I have, he felt that the Erelzi would be the most effective. He said that, in most cases, there weren’t any real problems, any real transition issues, and that it was just as effective from what he had seen at that time. He also said that if, “If you found that you were degrading, we would get you back on the original.”*	The support from healthcare providers throughout the switch to biosimilars
Lack of support	*“Nobody’s told me that I shouldn’t have [Inflectra] again*	Some participants shared their experience of lack of support during the switch, including lack of follow-up phone calls from the nurse
Theme 5B: effect of COVID-19 Pandemic on switch		
Impact on mental and physical health	*“[I’m] a type-A personality, so the COVID protocols have been more of an issue for me because everything around me has changed, not so much that I have changed. My life has settled into a quiet routine of just myself and my two dogs living in a little rental suite, so that hasn’t changed, but so much has changed around me.”*	Participants shared an increase in feelings of anxiety during the pandemic, as well as changes to their ability to perform physical activity during the lockdowns
Changes to receiving and delivery of healthcare	*“[Telehealth is] a little different, but I mean, it’s, it works for me because, I mean, other than they don’t get to touch my joints to see if they’re swollen or things like that, but I can, I’ve had this long enough that I’m able to tell them what I’m feeling and what’s going on.”*	Participants expressed changes to the way they were receiving care, particularly an increased difficulty in making appointments; however, there was an increase in the use of telehealth for patient appointments

During these interviews, participants were asked to reflect back on their baseline opinions and attitudes prior to the switch (Theme 1B), where they shared negative expectations, apprehension, and concerns surrounding disease control. During the switch, participants shared that their experienced enablers (Theme 2B) included their informed acceptance of the policy change, successful management of their medical condition throughout the changeover, as well as the limited adverse effects experienced. Cumulatively, these factors made for an overall positive experience for the majority of the participants. Participants also shared experienced barriers of the switch (Theme 3B) including some participants who experienced adverse effects (e.g., discomfort or pain at injection site) which were potentially attributed to the change in needle type from the biologics. Collectively, these negative experiences led some participants to express the desire to revert to their originator drug. Participants also shared the presence of their support systems (Theme 4B) made up from family, friends, and their healthcare providers. One participant shared that they “really [trusted] and [appreciated] the informed atmosphere” from which their rheumatologist was operating.

The timing of the switch coincided with the onset of the global COVID-19 pandemic, and participants described impacts on their mental health, namely, an increase in feelings of anxiety. There were also changes, secondary to the pandemic, in the way participants received healthcare and the delivery mechanisms of this care. Whilst participants experienced difficulty physically seeing rheumatologists and making appointments, they reported an increase in the use of telehealth.

### Thematic map of major themes from participants post-switch

**Fig. 2:**
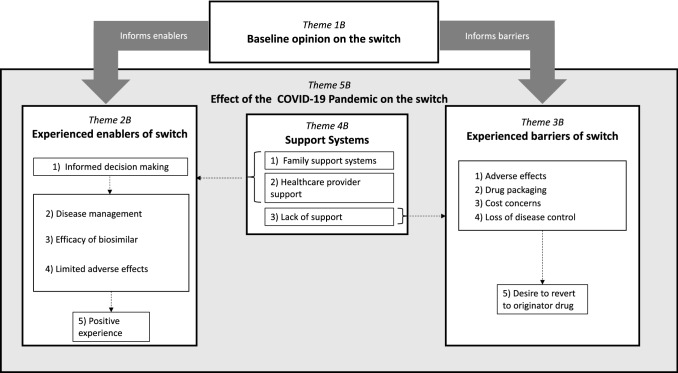
A thematic map depicting how a patient’s baseline opinion of the switch (Theme 1B) informs both their experienced enablers (Theme 2B) and barriers (Theme 3B) of the switch. These themes are encompassed by the overarching impact of the COVID-19 pandemic that was concurrent to the switch (Theme 5B). Dashed arrows show relationships between and within sub-themes

Relationships between and within themes drawn from interviews post-switch are depicted in Fig. 2. Participants’ baseline opinion on the switch (Theme 1B) informs both enablers (Theme 2B) and barriers (Theme 3B) experienced by participants during the policy change. Participants’ informed decisions regarding the switch may be linked with their ability to manage their disease, their experiences of efficacy from the biosimilar, and the minimal adverse effects experienced. Conversely, barriers to the policy change, including an experience of adverse effects, changes to the drug packaging and/or administration, cost concerns, and a loss of disease control reinforced participants’ desire to revert to their originator drug. Both experienced enablers (Theme 2B) and barriers (Theme 3B) are influenced by participants’ support systems (Theme 4B). Specifically, the presence of support from healthcare providers or family members contributing to the enablers, while lack of support contributing to barriers of the switch. The effect of the COVID-19 pandemic on the switch (Theme 5B) encompasses the participants overall experience, affecting participants’ enablers (Theme 2B), barriers (Theme 3B), and support systems (Theme 4B).

## Discussion

With the increasing interest in biosimilar use and non-medical switching policies worldwide, capturing and understanding patient perspectives before, during, and after the switch is integral to future policy decision-making and understanding the impact these changes have had on affected patients. Patient perspectives on similar policy changes have been characterized in the past; however, to our knowledge, this is the first study examining and characterizing the Canadian patient perspective both pre and post-implementation of this novel policy change in North America [18, 19]. This study characterizes the patient perspective on the BC Biosimilars Initiative both before and after their switch to biosimilars. The findings of this study emphasize the apprehension and anxiety participants experienced prior to the switch, while capturing their successful changeover to a biosimilar with the support from their healthcare providers and families.

Particularly, prior to their switch, one of the most common concerns from participants was the potential decrease or lack of efficacy and increase in side effects of the new biosimilar agent. This is consistent with findings from a patient survey in the United States conducted by Teeple et al. that estimated that 85% of their respondents were concerned with a decrease in efficacy of the biosimilar and worried it would not treat their condition as well as their corresponding biologic agent. Further, 83% of their respondents were also concerned that the biosimilar would lead to an increase in side effects [18]. Similarly, the majority of participants in a 2019 French survey conducted by Frantzen and colleagues shared concerns for biosimilar efficacy and safety profiles when compared to their originator drug [19]. Results from our study show similar concerns and patient apprehension towards biosimilar prior to their switch.

All participants in this study expressed a good baseline understanding of the similarities and differences between biologics and biosimilar, the rationale for the policy change, and were informed of this change prior to the implementation of the switch. This contrasts with findings from Teeple and colleagues who found that 64% of participants had no knowledge at all regarding biosimilars and findings from Frantzen and colleagues who found that 44% of participants were not informed about their changeover to biosimilars [18, 19]. Evidently, the knowledge and information shared to patients is varied across practices; however, despite our patient population’s knowledge base, participants still approached their switch to biosimilars with apprehension and anxiety.

Proper and effective communication strategies from healthcare providers to patients regarding the switch is integral to the success of their changeover and disease management. The importance of effective patient communication has been emphasized to prevent the occurrence of the ‘nocebo’ effect, defined as the “worsening of symptoms induced by any negative attitude from a non-pharmacological therapeutic intervention” [20, 21]. When patients approach the drug with a negative attitude and apprehension, they may experience a lack of or decrease in efficacy [20]. For example, results from the 2017 NOR-SWITCH trial examining the switch from originator infliximab to its corresponding biosimilar (CT-P13) found that disease worsening occurred in both biologic and biosimilar arms of the study [22]. Kristensen and colleagues have hypothesized that the findings from the NOR-SWITCH trial may have been a result of the nocebo effect [20]. Though the majority of participants in our study approached the policy change with fear and apprehension, participants shared in post-switch interviews that their support systems including their rheumatologist, pharmacist, or social circles had facilitated a smooth transition for them. Though previous research has shown that patients received varied information of the switch, including a lack of notification prior to their change, participants from our study shared that they received and appreciated the timely information provided to them by their healthcare providers prior to the policy change [18, 19]. The open communication, early notification, and support from rheumatologists in this study may have contributed to the success of participants’ switch to biosimilars and absence of an observed ‘nocebo’ effect.

In light of the COVID-19 global pandemic that spanned the duration of this policy change, the impact of the pandemic on patients’ biosimilar experiences was also characterized as part of this study. It is unsurprising that the onset of a widespread pandemic would have a significant impact on the emotional wellbeing of individuals [23]. The psychosocial impact of the COVID-19 pandemic can be substantial; an early study of the impact found that 54% of individuals reported the psychological impact of the pandemic as moderate to severe [24]. Participants from our study shared similar psychosocial impacts of the pandemic, namely, an increase in feelings of anxiety or depression, which may have contributed to their apprehension and fears going into the switch. Nevertheless, with the decrease in in-person rheumatologist visits, participants reported an increase in their use of telehealth to facilitate their switch. With the advent of telemedicine, telehealth has provided the opportunity for patients to maintain their continuity of care while limiting their exposure to infection [25]. The growth of the use of telehealth, particularly for this cohort, were met with its own barriers and enablers. Barriers to telehealth included the additional planning required to set up technology and lack of physical examinations. Enablers to telehealth included the ease and convenience for patient appointments, as well as a smooth transition facilitated by care providers over to the new platform. In general, participants in this study spoke to the convenience of this health delivery model and for the most part, their seamless transition to a biosimilar during a global pandemic.

Our study does have limitations. Although our sample size was small, using convenience sampling rather than thematic saturation to determine participant numbers, and we only recruited from two practices, we believe that we were able to achieve thematic saturation through our qualitative analysis. We were able to capture patient perspectives from urban areas and more rural settings, which can mean findings are informative for similar patient populations. The data collected in this study achieved variability in terms of participant demographics and geographical location. The recruiting clinics sought to invite patients with diverse perspectives on mandatory switching. Further, not all participants took part in a follow-up interview and thus post-switch results may not have been fully or accurately characterized. Lastly, responder bias may have occurred as individuals who participated in these interviews may have been more inherently engaged in their medication management and their transition to biosimilars, or alternatively had a stronger sentiment against mandatory switching. However, this diversity of perspective is valuable for qualitative research. Despite these limitations, our study is the first of its kind to characterize both pre and post-policy change patient perspectives during a limited window of opportunity ahead of the first mandatory switching policy in Canada. Therefore, this qualitative study of patient perspectives of the BC Biosimilars Initiative adds to the growing body of literature surrounding patient experiences of non-medical switching to biosimilars. Future research will be needed to build on our findings to explore the impact of the BC Biosimilars Initiative on physician prescribing patterns, patient outcomes, and the intended and any unintended consequences related to drug utilization and costs for payers in the province.

## Conclusion

The results from or study characterize the patient perspective both pre- and post-implementation of a top-down, province-wide policy change and illustrate how patient concerns prior to such policy changes can be addressed with adequate patient-provider communication and support, leading to patient satisfaction and adequate disease management post-switch. Participants’ perspectives shown in this study can help to inform implementation methods for future policy changes of a similar nature. The support from social circles and healthcare providers experienced by participants as well as the shared decision-making facilitated a smooth transition from biologics to biosimilars, despite the apprehension and anxiety of participants prior to their switch. Communication could be improved in the future if policy makers can anticipate some of the fears and expectations of patients as seen in this study. Future studies with larger samples could help characterize the patient experience on a wider scale and allow for more generalizable findings and applicability to policy makers.

## Data Availability

The datasets used and/or analysed during the current study are available from the corresponding author upon reasonable request.
